# Report from a Tibetan Monastery: EEG neural correlates of concentrative and analytical meditation

**DOI:** 10.3389/fpsyg.2024.1348317

**Published:** 2024-05-02

**Authors:** Bruno Neri, Alejandro Luis Callara, Nicola Vanello, Danilo Menicucci, Andrea Zaccaro, Andrea Piarulli, Marco Laurino, Ngawang Norbu, Jampa Kechok, Ngawang Sherab, Angelo Gemignani

**Affiliations:** ^1^Dipartimento di Ingegneria dell’Informazione, University of Pisa, Pisa, Italy; ^2^Research Center “E. Piaggio”, University of Pisa, Pisa, Italy; ^3^Department of Surgical, Medical and Molecular Pathology and Critical Care Medicine, University of Pisa, Pisa, Italy; ^4^Department of Neuroscience, Imaging and Clinical Sciences, “G. d’Annunzio” University of Chieti-Pescara, Chieti, Italy; ^5^Istituto di Fisiologia Clinica, CNR, Pisa, Italy; ^6^Sera Jey Monastic University for Advanced Buddhist Studies & Practice, Bylakuppe, Mysore, India

**Keywords:** analytical meditation, concentrative meditation, Buddhist monks, EEG, neural correlates

## Abstract

The positive effects of meditation on human wellbeing are indisputable, ranging from emotion regulation improvement to stress reduction and present-moment awareness enhancement. Changes in brain activity regulate and support these phenomena. However, the heterogeneity of meditation practices and their cultural background, as well as their poor categorization limit the generalization of results to all types of meditation. Here, we took advantage of a collaboration with the very singular and precious community of the Monks and Geshes of the Tibetan University of Sera-Jey in India to study the neural correlates of the two main types of meditation recognized in Tibetan Buddhism, namely concentrative and analytical meditation. Twenty-three meditators with different levels of expertise underwent to an ecological (i.e., within the monastery) EEG acquisition consisting of an analytical and/or concentrative meditation session at “their best,” and with the only constraint of performing a 5-min-long baseline at the beginning of the session. Time-varying power-spectral-density estimates of each session were compared against the baseline (i.e., within session) and between conditions (i.e., analytical vs. concentrative). Our results showed that concentrative meditation elicited more numerous and marked changes in the EEG power compared to analytical meditation, and mainly in the form of an increase in the theta, alpha and beta frequency ranges. Moreover, the full immersion in the Monastery life allowed to share the results and discuss their interpretation with the best scholars of the Monastic University, ensuring the identification of the most expert meditators, as well as to highlight better the differences between the different types of meditation practiced by each of them.

## 1 Introduction

*Meditation* refers to a set of highly differentiated practices that, through specific mental training, improve physiological functions and self-regulating abilities of practitioners, as well as their cognitive, emotional, and spiritual aspects (Van Dam et al., 2018). Meditative practices are at the basis of Eastern traditions, cultures and philosophies, such as Buddhism and Hinduism, which describe meditation mainly through its mental and mystical facets. In the Western culture, the study of meditation is tightly related to the concept of mindfulness. This term was defined by John Kabat-Zinn as “the awareness that arises by intentionally paying attention, in the present moment, to the flow of experience from moment to moment, in a non-judgmental way” (Kabat-Zinn, 1991). To reach the mindful state, a set of contemplative practices derived from the Buddhist tradition, such as Samatha and Vipassana, have been simplified and standardized to be applied in secular and clinical contexts such as the psychotherapy of depression and anxiety disorders, as well as stress reduction (Vago and Silbersweig, 2012; Farb et al., 2015; Jerath et al., 2015).

Investigating mindfulness and meditation practices from a western perspective is complex, since the plethora of these practices is vast and heterogeneous (Fox et al., 2016; Fox and Rael Cahn, 2021). However, there are some elements common to the different meditation traditions, which have been empirically classified by the Contemplative Neuroscience field (Lutz et al., 2008; Nash et al., 2013; Dahl et al., 2015). An influential classification of meditative practices was first proposed by Lutz et al. (2008), who defined two macro-areas: (1) Open Monitoring Meditation (OMM), which involves non-reactive monitoring of the contents of experience; and (2) Focused Attention Meditation (FAM), which entails the voluntary focusing of attention on a chosen object (usually the breath). Both practices are widely used in mindfulness-based protocols, and involve the ability to intentionally activate, direct and sustain various attention processes, hence strengthening the ability to be aware of thought processes, emotions and the body (Lutz et al., 2008).

The OMM does not require focusing on a specific object but is characterized by the expansion of the attention range to the whole external environment and to the internal mental space, to become aware of every perception, thought and emotion present in the field of awareness. Therefore, the meditator remains receptive to elements that gradually enter the field of awareness, although non-reactive toward them, avoiding cognitive interpretations and emotional responses (Dahl et al., 2015). OMM can be a used as preparatory step for other types of meditation and was associated with activations of crucial regions for body awareness, interoception, and attention monitoring such as the inferior frontal gyrus, the pre-motor cortex, the dorsolateral prefrontal cortex, the anterior dorsal cingulate cortex and the insula, as shown by a meta-analysis of functional Magnetic Resonance Imaging (fMRI) studies (Fox et al., 2016). Significant deactivations were also observed in the thalamus, a structure involved in the selective filtering of sensory signals arriving from the environment, which may lead to an open and receptive state to sensory stimuli (Fox et al., 2016).

FAM in Tibetan Buddhism is called Concentrative Meditation and represents, in such tradition, one of the two main forms of meditation, together with analytical meditation (Dalai Lama, 1995; Wallace, 1999). It is the most studied form of meditation, due to its straightforwardness. The meditator initially focuses the attention on the breath (or another object, for instance the recitation of mantra) and, each time the mind gets distracted and starts wandering from one thought to another (i.e., mind wandering; see Buckner and Carroll, 2007), the meditator has to become aware of it, detach from the contents of the distractors, and gently refocus the attention on the breath (Wallace, 2006). With practice, distractors will become more rarefied, and the mind will be able to focus on the object, reaching deeper states of concentrative absorption characterized by calm and mental stability. When this state is stable and naturally maintained without effort, it is called Shamatha.

Some consensus is being reached regarding the activity and connectivity of specific cortical networks during Concentrative meditation, mainly using fMRI and Electroencephalogram (EEG) studies (Cahn and Polich, 2006; Fox et al., 2014; Tang et al., 2015; Fox et al., 2016). In this context, the so-called Default Mode Network assumes great importance. It includes the medial prefrontal cortex, the posterior cingulate cortex, the posterior inferior parietal lobule, and the precuneus, and has been associated to verbal thinking concerning the self, autobiographical memory, introspection and mind-wandering, but also to rumination, stress, and depression (Brewer et al., 2011; Raichle, 2015). An important meta-analysis of fMRI studies (Fox et al., 2016) revealed that Concentrative meditation increases the activity of brain regions associated to cognitive and attention control. Activations were reported in the premotor cortex, dorsolateral prefrontal cortex, and anterior dorsal cingulate cortex. In addition, deactivations were reported in the posterior cingulate cortex and posterior inferior parietal lobule, main nodes of the Default Mode Network (Fox et al., 2016). These data suggest that Concentrative meditation can reduce spontaneous thoughts about past and future events, leading to a state of consciousness focused on the present moment (Fox et al., 2016). At the EEG level, coherent findings revealed that Concentrative meditation is related to enhanced alpha and theta power, compared to a resting state condition, in both healthy individuals and patients (Lomas et al., 2015). More specifically, Concentrative meditation appeared to be related to increases of both anterior and posterior alpha power, and to fronto-temporal and parieto-occipital theta activity. Finally, it is also related to increased posterior gamma power, while no clear changes were found for beta activity (Lee et al., 2018). This co-presence of high gamma, alpha and theta activity may indicate a state of consciousness characterized by enhanced awareness, relaxed alertness and well-being (Lomas et al., 2015).

According to the Tibetan Tradition, starting from Shamatha, one can explore other meditative states, e.g., analytical or vipassana meditation. Analytical meditation consists of fixing the mind on a specific idea or concept, with concentration and steadiness. In the Tibetan Buddhism, analytical meditation is used to break down these ideas or concepts into their constituent parts. Therefore, the attentional focus cultivated through analytical meditation naturally includes the awareness of certain philosophical principles that support meditators’ ability to perceive reality (Mehrmann and Karmacharya, 2013). In addition, this practice requires a deep knowledge of the theoretical basis of the meditation, as well as the ability to keep the mind focused on a specific concept, such as the nature of reality, the meaning of Karma, reincarnation, the nature of impermanence, etc. In this sense, analytical meditation does not differ from Vipassana, which in Tibet is called Lhakthong, meaning “to see more,” or “to see beyond,” and is used to analyze and explore in depth the object of attention.

Despite the undeniable progress made by scientific research on contemplative practices, a clear convergence toward common conclusions is not observed in the literature, and there are still many outcomes and states of consciousness that have not been examined in the scientific literature. Some of the most pressing questions and concerns include the lack of clarity in the definition of mindfulness and meditation, the challenges of measuring meditation experience, the need for more diverse samples in meditation research, and the need for more research on the long-term effects of meditation. Additionally, there are debates regarding the efficacy and safety of meditation-based interventions, the potential for adverse effects of meditation such as increased anxiety, depression, or dissociation, as well as the potential for biases in meditation research such as exaggerated positive claims, often caused by public misunderstandings and inaccurate news media publicity (“mindfulness hype”) (Van Dam et al., 2018; Vieten et al., 2018). It is the authors’ opinion that one of the main problems arises when one tries to compare the objective results of the measurements with the meditator’s first-hand experience. This is because the narration of this experience and the terminology used depend on the context and the cultural and experiential background of the meditator. Recently, some studies aimed at reducing this gap by directly collaborating with monastic universities (Jiang et al., 2020; Lott et al., 2020; van Vugt et al., 2020; Medvedev et al., 2022). In this direction, one of the peculiar characteristics of this work, consists in the fact that all the volunteers who participated in the research (monks and Geshe of the Sera Jey Monastery) have the same cultural background and have shared for at least 10 years the life of the monastery. Furthermore, they have been examined without taking them out of their usual context.

Considering the scientific world’s growing interest in contemplative practices and taking note of the fact that, despite the huge number of publications on the subject, there are still several controversies and open issues (see Van Dam et al., 2018; Vieten et al., 2018), at the University of Pisa we decided to exploit a series of favorable conditions for attempting to make an original contribution to the exploration of this fascinating and still largely unknown territory. In 2016, at the suggestion of His Holiness the Dalai Lama, the Lama Tzong Khapa Institute of Pomaia (the largest center of Mahayana Buddhism in the West, about 35 min from Pisa) and the University of Pisa entered into a cooperation agreement in sectors of teaching, science and cultural dissemination on topics of common interest. Since many masters and resident Lamas of the ILTK had studied at Sera Jey, it was easy to enter into an agreement with the Sera Jey Monastic University as well. In this way the possibility of collaboration was also created with one of the most authoritative and ancient institutions of Tibetan Buddhism. The opportunity was unique, allowing us to work with a group of meditators belonging to the same tradition, with the same cultural background, and who share the same environment and the same habits of life in a stable and homogeneous context, fruit of a millenary tradition. In these conditions, the same definitions and the same taxonomy crystallized over the centuries constitute a common heritage, and it is also possible to have the voluntary collaboration of expert meditators, with tens of thousands of hours of meditative experience, without removing them from their environment.

Another peculiarity of the population of volunteers recruited for the research is the fact that, although they all came from the same context, it was made up of meditators with very different levels of experience: from beginners with less than 1 year of practice and, on average, 20/30 min of daily practice, to intermediates with a practice period of between 1 year and 10 years and 60/90 min of daily practice, up to advanced ones, who all with more than10 years of practice and at least 6 months of retreat exclusively dedicated to meditation. In this case, although there are differences depending on the type of retreat chosen by the volunteer, typically 4 daily meditation sessions lasting about 2 h are practiced interspersed with equally long periods of rest which correspond about 30,000 h in 10 years. All volunteers were experts in at least one of the two most practiced types of Meditation in Tibetan Buddhism in the Sutra system: Concentrative Meditation and Analytical Meditation.

In this study, we characterize the neurophysiological correlates of analytical and concentrative meditation by means of an EEG study in a cohort of beginners, intermediate and expert meditators from the Sera-Jey Tibetan Monastery. We hypothesized that both subject-specific peculiarities as well as different levels of expertise would result in meditation sessions of different durations to achieve specific mental states. In this light, we asked each volunteer to “do their best” with the only constraint of performing a 5-min closed-eye resting state baseline at the beginning of the meditation session. On these datasets, we perform several different analyses aiming at: (i) characterizing the changes in the time-varying EEG spectra underpinning analytical and concentrative meditation, (ii) identifying the key neural correlates distinguishing analytical and concentrative meditation, and (iii) exploring the influence of expertise on such differences.

## 2 Materials and methods

All the data acquisition was performed in a period of 12 weeks within the social, cultural and environmental context shared by all the volunteers for at least the last 10 years of their lives. A detailed description of such a context is given below.

### 2.1 Context: full immersion in a Tibetan Monastery

One of the characteristics of this work consists in the fact that the volunteers belong to a homogeneous sample both from the point of view of cultural and historical heritage and from that of habits and living environment. For this reason, we believe it is appropriate to devote space to the description of these two aspects.

Sera Jey Monastery was founded in the early 15th Century by Kunkhen Lodroe Rinchen Senge, at the time of Lama Tsong Khapa (1357–1419), founder of the Gelukpa School, the one to which also His Holiness the Dalai Lama belongs. In 1959, before the Chinese occupation, the number of monks exceeded 5,600. A few hundred people, including lamas, geshes and monks, managed to escape to India and moved to the settlement of Bylakuppe in the Mysore district of the state of Karnataka. The Sera Jey Monastery has been rebuilt and the community of monks currently numbers around 3,000 individuals. So, the unbroken lineage of masters dating back to the founder of the Gelukpa School survived and flourished again on the Indian plateau (about 1,000 m above sea level), covered by jungle. Monks and Geshe stay in Khangtsens, that are sections of the Monastery each one corresponding to one different region of Tibet and each new coming monk is placed in a specific Khangtsen according to his place of origin.

Sera Jey Secondary School and the monastic Education Board are affiliated to the Sera Jey Monastic University. The university offer course for 25 years and at the end of the curriculum, at the age of about 35, students are awarded the title of Geshe which is equivalent to a PhD in Buddhist Philosophy.

This monastic institute follows the pure and unbroken lineage of Buddha’s teachings and its classical commentarial works written by the eminent ancient Indian Buddhist scholars and practitioners of the prestigious Nalanda University in India. This Indian rich spiritual knowledge and practices has been spread and well flourished across the Asian countries through various mediums, the complete teachings of Buddha and the lineages of Nalanda spiritual treasure and practices were fully inherited by Tibetan master scholars in Tibetan language in the 7th century. The monastic curriculum primarily consists of memorization, dialectal debate, prayer and meditation. The monks study five major philosophical subjects based on classical Indian Buddhist texts. The five subjects are: (i) Valid Cognition (Pramana) to learn how to use valid reasoning in analyzing the texts; (ii) Perfect of Wisdom (Prajnaparaminta) to learn how to develop the realization of the path and grounds to enlightenment; (iii) The Midddle Way (Madyamika) to learn about the profound views of dependent origination and emptiness (Shunyata); (iv) Monastic Discipline (Vinaya) to learn how to live moral life as monastic rules and regulation set out by Buddha; and (v) Phenomenology (Abhidharma) to learn different aspect of mind and mental factors and theory of karma. To study the above major subjects in depth and details to earn final degree in Buddhist philosophy, the monastic Board of Education laid out the complete syllabus based on the scriptural texts and the commentarial works of Tsongkhapa, the founder of Gelugpa School, and his disciples on the classical works of eminent Indian Buddhist scholars of Nalanda University (developed between the 1st and 11th centuries) such as Nagarjuna, Dharmakirti, Dignaga, Arya Asanga, Chandrakirti and Visubhandu.

The most gifted ones can continue for another 6 years and obtain the title of Geshe Larampa. Graduates Geshes then join nearby Tantric College of Gyumed to study tantric and esoteric meditative practices for 1 year. Some graduate Geshe enter the monastic and meditation and retreat center where they put into practice spiritual knowledge by performing ritual and contemplative meditation for several years codified protocols of meditative practices, unchanged for centuries, accumulating tens of thousands of hours of practice. They dedicate oneself to meditation in a structured way after having completed their studies. The others highly learned Geshes remain in the monasteries to teach or contribute to the life of the Monastery and the University or assigned to teach Buddhist philosophy and doctrine in India, Nepal, Bhutan, Taiwan and the rest of the world where there are several Tibetan culture centers linked to Sera Jey.

A Committee for Sera Jey Philosophical Studies & Board of Examination is responsible for the administration of Monastic University courses, and Sera Jey Modern Education Department holds a similar responsibility that of a modern Academic University Department.

### 2.2 Participants

Twenty-three Geshes and monks (all males) from the Sera Jey Monastery took part in the experiment. All subjects gave their written informed consent. The studies involving humans were approved by ethical committee of the University of Pisa (n. 0117745/2020). The studies were conducted in accordance with the local legislation and institutional requirements. The participants provided their written informed consent to participate in this study. The data collection took place over the course of three stays at the Sera Jey Monastery, whose overall duration was approximately 12 weeks. This allowed acquiring data from the participants in their own cultural environment, aiming at limiting potential uncomfortable conditions for the meditators. Details on the subject’s population are reported in Table 1. We divided the participants into three classes based on their level of experience. Nine participants were considered beginners -B- (i.e., 20/30 min of daily practice and less than 1 year of experience), 6 intermediate -I- (i.e., 60/90 min of daily practice and between 1 year and 10 years of experience) and 8 advanced -A- (i.e., full-time meditators with at least 6 months of retreat).

**Table 1:** Study population.

ID	Category	Age	Retreat periods (years)	N. of concentrative sessions	N. of analytical sessions
1	A	54	5	1	0
2	A	64	7	4	0
3	A	51	13	1	1
4	A	55	5	3	0
5	A	50	2	1	1
6	A	40	0.5	1	1
7	I	78	N/A	1	1
8	A	78	9	1	0
9	I	37	N/A	1	1
10	A	53	11	1	1
11	B	36	N/A	1	0
12	I	34	N/A	0	1
13	B	39	N/A	0	1
14	B	36	N/A	0	1
15	I	41	N/A	0	2
16	B	29	N/A	0	1
17	I	50	N/A	0	1
18	B	39	N/A	0	1
19	B	39	N/A	0	1
20	B	31	N/A	0	1
21	B	36	N/A	0	1
22	I	46	N/A	0	1
23	B	34	N/A	0	1

For each subject ID, category, age, periods of retreat and number of sessions performed for each type of meditation are reported. Category describes the experience of the subject and can be among A (advanced), I (intermediate), B (beginners). N/A, not applicable.

### 2.3 Experimental procedure

Before starting the experiment, participants filled in a questionnaire reporting their level of experience, the type of meditation usually practiced, the reference system (tantra or sutra) and whether they have been on retreat. Details on the questionnaire are reported in the Supplementary materials (ms_neri_et_al_frontHumNeu_supp_mat_questionnaire.docx).

Then, the experiment began, and participants were asked to perform analytical and/or concentrative meditation sessions, under the sutra system.

Briefly, concentrative meditation (shamata) involves focusing and sustaining attention on a single object, for example the breath or a point of visual fixation or a mantra. This allows the mind to calm down and eliminate noises and discursive thoughts, and to reach the so-called “calm abiding” (Tibetan: shinè), a state of pure awareness without content. This corresponds to a calm and concentrated mind and to a suspension of conceptual thought. This state can also be used as a starting condition for other types of meditation used to investigate, for example, the roots of the mind–body relationship.

Instead, analytical meditation is a form of meditation in which the meditator initially directs his/her attention to what appears to the mind without attachment and by cultivating awareness. Then, the meditator focuses and analyzes in depth a particular concept of the Dharma (e.g., emptiness, compassion, lamrim, consciousness and its levels). As a result, this practice corresponds to a clear mind and is well described as a logic/conceptual task. This mental state can be also reached by practitioners during the so-called Tibetan debate, which consists in a verbal challenge between two experts on Buddhist philosophy and Buddha teachings (van Vugt et al., 2020), although debate is not needed for performing analytical meditation.

Meditators were invited to commit themselves as fully as possible to the chosen practice and left free to “do their best” without time limits, but with the only constraint of performing a 5 min long resting-state baseline before the session. Moreover, to allow everyone to do their best without external forcing, meditators were also allowed to retire to another room during the meditation session. Within these heterogeneous datasets, we aimed at identifying the greatest deviations from the resting state baseline. Accordingly, since we did not have detailed information on the various phases of the sessions, the entire session was studied. At the end of the session, the volunteer underwent a debriefing about meditation (reported in the questionnaire).

### 2.4 Physiological data acquisition

We acquired EEG signals using a portable electroencephalograph with 19 channels according to the 10–20 international standard (EBNeuro BE PLUS LTM, Florence – Italy). Signals were acquired at a sampling rate of 512 Hz. The system was provided with a special shoulder bag for the portable recorder, allowing the subjects to freely move during the experimental session (e.g., retiring in a quiet place). The subject was prepared with preliminary scalp scrubbing before wearing the EEG cap. The contact resistance of all the electrodes was measured before the start of the experiment and kept below 25 KW throughout the recording. At the end of the experiment, contact resistances were checked again to handle possible non-negligible differences in the impedances.

### 2.5 Physiological data analysis

#### 2.5.1 Preprocessing

EEG signals were downsampled to 100 Hz, after applying a low-pass antialiasing filter (cut-off frequency = 45 Hz). The obtained signals were high-pass filtered at 1 Hz with zero-phase non-causal filter. Then, signals were examined for the presence of bad channels through a two-step semi-automatic procedure. First, those channels whose correlation with the reconstructed version obtained from their neighbors was lower than a predetermined threshold (here set to a value of *ρ* = 0.7) (Mullen et al., 2015) were removed. Then, the EEG signals were visually inspected to check for the presence of potential noisy channels not captured by the correlation criterion (Urigüen and Garcia-Zapirain, 2015; Billeci et al., 2023). Removed channels were recovered through spherical spline interpolation (Delorme and Makeig, 2004). Afterwards, EEG signals were re-referenced to the average of all channels and analyzed with independent component analysis (ICA) using the AMICA algorithm (Palmer et al., 2011). ICA allows to decompose the EEG signals into sets of (maximally) statistically independent components, which represent independent sources of brain activity as well as different kinds of artifacts (e.g., muscular, ocular, channel noise). This procedure was found to be particularly suited for cleaning the EEG signal on the scalp by reconstructing it discarding the artefactual components (Urigüen and Garcia-Zapirain, 2015). Moreover, the interpretation of brain activity-related components was found to be particularly advantageous in terms of interpretability of brain sources in low density recording systems (Callara et al., 2020). Here, we reconstructed the activity on the scalp by removing the contribution of artifact-related components. All steps were performed using EEGLAB (Delorme and Makeig, 2004), and MATLAB custom scripts.

#### 2.5.2 Time-varying power spectral density analysis

For each subject, we obtained time-varying estimates of the power spectral density (tvPSD) of EEG signals using a sliding-window approach with 5-min-long non-overlapping windows (i.e., dividing the recording in 5 min consecutive time intervals and performing a PSD analysis for each of them). Within each of these windows, PSD was estimated using Welch’s overlapped segment averaging estimator on 5 s long consecutive windows, with 80% overlap and an imposing a zero-padding equal to 1,024 to improve PSD visualization. Finally, PSD was expressed in dB.

#### 2.5.3 Feature extraction

Starting from the tvPSD estimates, we extracted two main sets of features of interest: Average Frequency Band (AFB) PSD features and Continuous High Resolution (CHR) PSD (Δf = 0.0488 Hz) features. AFB PSD features were obtained for each electrode by taking the average tvPSD in the following bands: δ (1–4 Hz), θ (4–8 Hz), α_1_ (8–10 Hz), α_2_ (10–12 Hz), α_3_ (12–14 Hz), β_1_ (14–20 Hz), β_2_ (20–30 Hz), and γ (30–45 Hz). From these averages, we derived a global index of tvPSD by averaging it over all the electrodes (hereinafter called global PSD). Accordingly, we obtained a total of 8 features whose dynamics during the meditation session were analyzed by comparing their values with respect to the resting-state baseline.

CHR PSD features were designed to describe the time evolution of PSD within each recording. Specifically, they allow to explore, frequency by frequency, the PSD changes across each frequency band that could be masked by the averaging process performed to estimate AFB PSD. The features describe maximal positive (Δ+) and negative (Δ-) changes with respect to basal condition of the tvPSD across different frequency bands. In addition, the maximum variation of the Alpha peak with respect to basal condition was considered (Δ_α-peak_). This last feature was obtained by visually inspecting the spectrum and taking the maximum of the CHR PSD in the α band for each 5-min-long window. From a visual inspection of the CHR PSD, we noted the presence of a marked peak in the spectrum in the β range (hereinafter called *bump*) in several EEG traces. We extracted the number of sessions in which they were present, the difference between their maximum and its value during the baseline (Δ*_bump_*) and the frequency at which they occurred.

### 2.6 Effect of years of retreat on EEG neural correlates

The maximum variation of the alpha peak with respect to basal conditions (Δ_α-peak_) was further used to investigate the effect of the years in retreat of advanced meditators on EEG neural correlates. We focused on this feature since it was the one that showed the most marked differences between the two types of meditation (see section 2.9), and given its possible relationship with inhibitory attentional mechanisms (Foxe and Snyder, 2011; Hanslmayr et al., 2011; Diepen et al., 2019; Antonov et al., 2020). This analysis was performed on a smaller sample made of 4 monks performing a total of 11 sessions (mean = 2.2 sessions each monk). We decided to limit the analysis to these 4 monks to have rigorous control over the time spent in meditation. Indeed, all of them had been engaged for a minimum of 6 months to a maximum of 7 years in the same type of retreat (Yamantaka retreat), which involves a very specific number of meditation sessions (i.e., 4 per day), lasting approximately 2 h each.

### 2.7 Statistical analysis

Because of the great heterogeneity of the dataset in terms of session variability (i.e., subjects “did their best” with no specific constraints on the duration of the meditation session), we performed both within session statistics to quantify the evolution of the EEG spectrum within the meditation session, and between session statistics to compare the two types of meditations.

Within-session statistics were performed separately for each subject. Particularly, we evaluated significant differences between each 5-min long window of the meditation session and the resting baseline. To this aim, for each AFB PSD feature, we used a Wilcoxon rank sum test to test the null hypothesis of no significant difference between conditions (*α* = 0.05). Multiple hypothesis testing was controlled with the Benjamini-Yekutieli false-discovery-rate (FDR) method, which controls the level of false positives under the presence of a (generic) dependency structure among tests (Benjamini and Yekutieli, 2001). Based on this analysis, we extracted descriptive statistics of each type of meditation by quantifying the percentage of windows in which AFB PSD significantly differed from the baseline.

Between session statistics were performed at the group level on CHR PSD features. To this aim, we used linear mixed models (s) as implemented in the fitlme MATLAB function. These models allowed to handle the heterogeneity of our dataset, i.e., (i) the partially unpaired data (i.e., some subjects performing only one type of mediation and some others performing both) and (ii) the unbalanced number of observations (i.e., some subjects performing more than one session). For each feature, we fitted a specific model as in Equation (1), with ${\mathrm{meditation}}_{\mathrm{type}}$ as fixed factor and random intercept. Multiple hypothesis testing was controlled with the FDR method (Benjamini and Yekutieli, 2001).


(1) $$\mathrm{feat}\sim 1+{\mathrm{meditation}}_{\mathrm{type}}+\left(1|\mathrm{id}\right)$$

Finally, to investigate the effect of the years spent in retreat of advanced meditators on the maximum variation of the alpha peak, we used the model in Equation (2) with time:in:retreat as fixed factor and random intercept.


(2) $$\Delta_{\alpha -\mathrm{peak}}\sim 1+\mathrm{time}:\mathrm{in}:\mathrm{retreat}+\left(1|\mathrm{id}\right)$$

## 3 Results

We gathered data from 23 subjects performing analytical and/or concentrative meditation for a total of 35 sessions. Sixteen of these sessions were concentrative meditations, while the remaining 19 were analytical.

### 3.1 Within session changes in tvPSD of analytical and concentrative meditation

**Figure 1:**
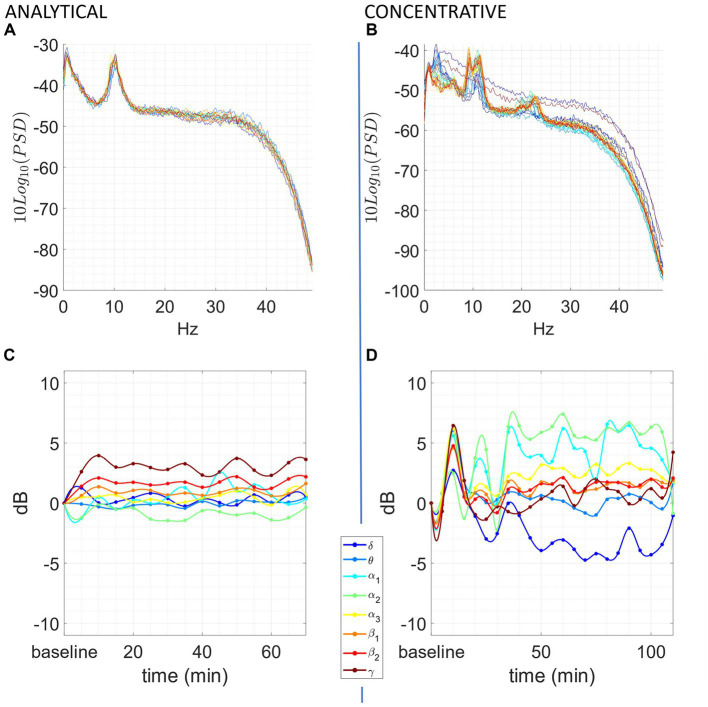
Exemplary of an analytical meditation session (left) and concentrative meditation session (right). Top: **(A,B)** tvPSD: each line in the spectrum corresponds to the PSD estimated in a 5-min-long window. Time is encoded in line color, from the beginning of the session (blue) to the end of the session (red). Bottom: **(C,D)** changes in tvPSD compared to baseline. The tvPSD is integrated in each considered frequency band.

We observed significant differences during each meditation session compared to a resting baseline. Most of the observable differences occurred during the concentrative sessions, although changes were present also in some of the analytic sessions. A prototypical example from the same participant of the changes elicited by analytical and concentrative sessions on tvPSD is reported in Figure 1, while single subject statistics are reported for each participant in the Supplementary materials (single_subject_ranksum_tests_results.zip). Particularly, for each subject we report the time windows during which AFB PSD features significantly differed from the baseline.

For the concentrative meditations, we observed that most changes emerge after 20/25 min from the beginning of the session and that these last until the late stages of the meditation session (an exemplary subject of this phenomenon is reported in Figure 1D). This effect which seems to be replicable across expert meditators, should be confirmed/refuted in intermediates by enlarging the sample size for this last group. For the analytical meditation, changes were less evident compared to the concentrative sessions.

**Figure 2:**
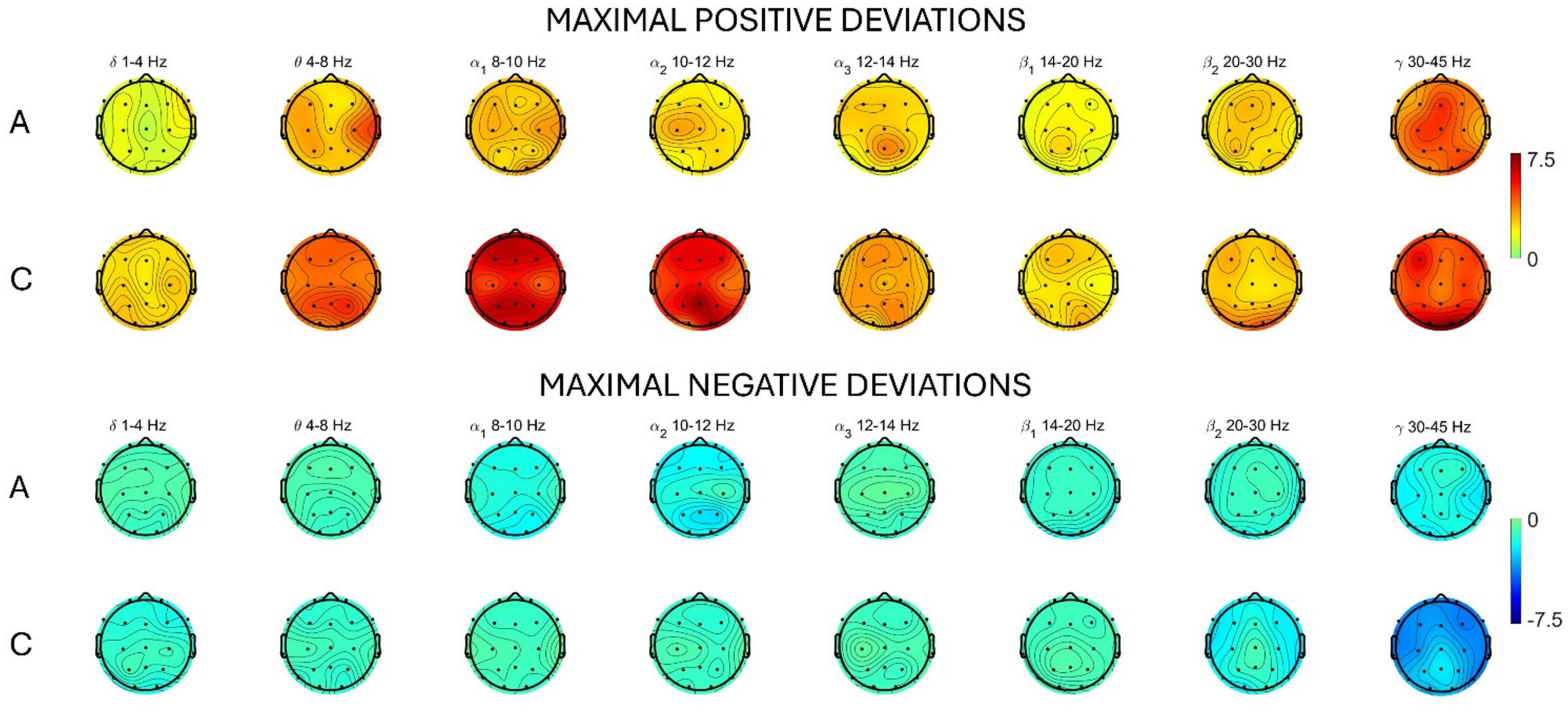
Average scalp map distribution for analytical (A) and concentrative (C) meditation. For each frequency band, the maximum difference (dB) (averaged across subjects) between the PSD during the session and the baseline is reported. Top: maximal positive deviations from baseline. Bottom: maximal negative deviations from baseline.

In Table 2, we summarize the within-subject analysis by reporting, for both types of meditation, the percentage of windows during the session that significantly differed from the baseline. We observed that concentrative meditation induced more changes in the EEG spectrum compared to analytical meditation in all frequency bands except for β_1_, for which, changes were comparable between meditative practices. Yet, both types of meditation produced significant changes compared to the baseline. A scalp distribution of maximal positive and negative differences observed during the meditation session is reported for each type of meditation in Figure 2. Concentrative meditation showed most marked changes in positive deviations from the baseline compared to analytical meditation. Among these, changes were mostly observed in α band for frontal and posterior regions. Similarly, most marked changes in negative deviations from the baseline were observed for concentrative sessions, mainly in the γ band for frontal regions.

**Table 2:** Percentage of windows with significantly different tvPSD with respect to baseline for each frequency band.

	Analytical	Concentrative
δ	33.33%	58.33%
θ	50.00%	66.67%
α_1_	71.43%	86.36%
α_2_	50.00%	66.67%
α_3_	28.57%	80.00%
β_1_	71.43%	68.18%
β_2_	71.43%	77.78%
γ	76.92%	83.33%

### 3.2 Neural correlates of analytical and concentrative meditation

Analytical and concentrative meditation were also compared directly, by using LMMs. The aim was to identify the features that significantly differed between the two types of meditation, as well as to provide robust EEG measures that best characterized such differences.

**Figure 3:**
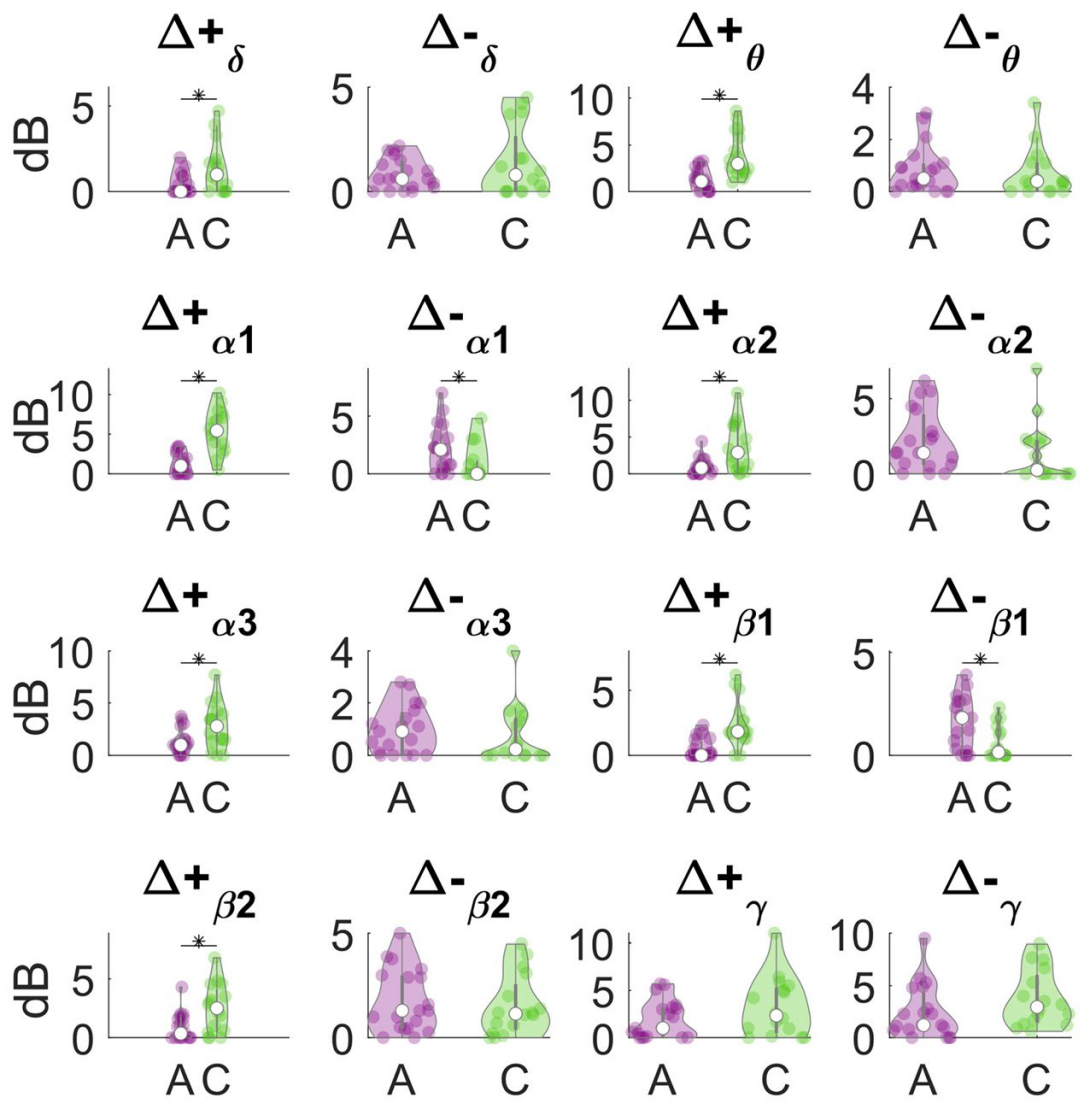
Analytical vs. concentrative. Violin plots of frequency-band deviation in dynamic PSD. Statistically significant differences (*p* < 0.05, FDR- corrected) are marked with an asterisk (*).

**Figure 4:**
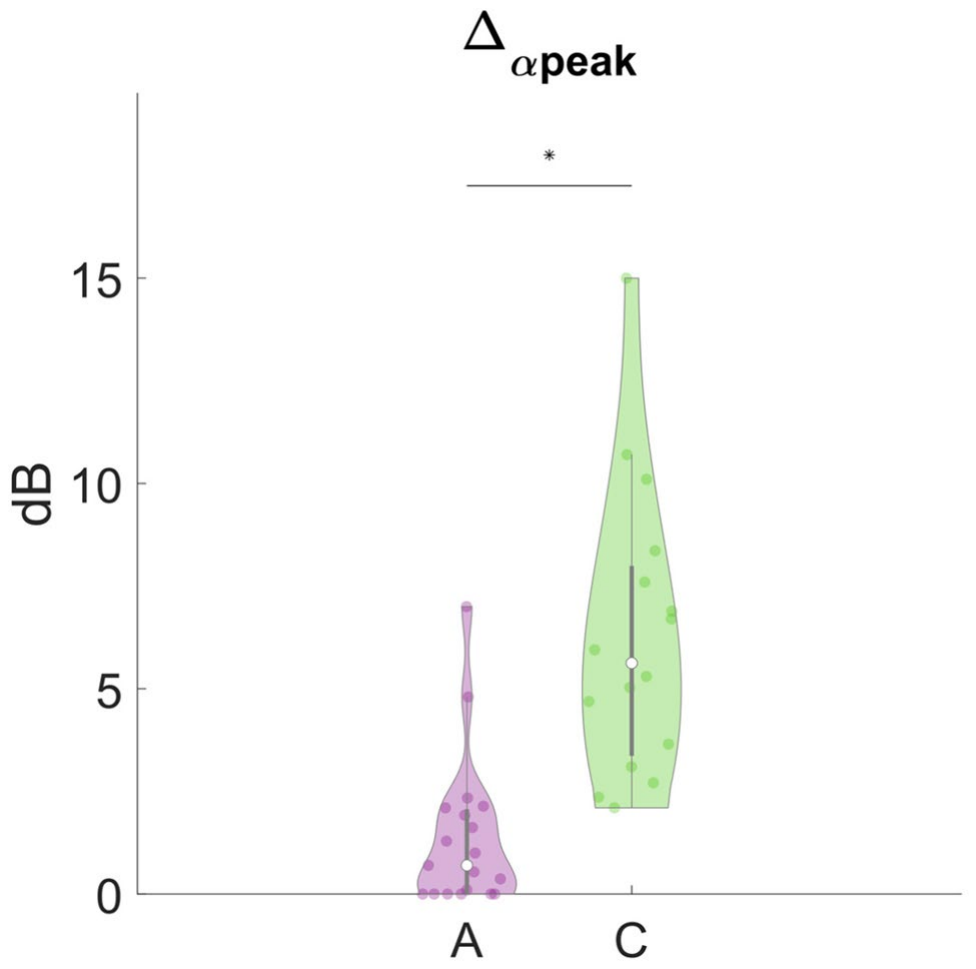
Analytical vs. concentrative. Violin plots of Δ_α-peak_. This feature was the feature with the most marked differences between analytical and concentrative sessions. Statistically significant differences (*p* < 0.05, FDR- corrected) are marked with an asterisk (*).

In Figure 3, we report the results of the main effects of the type of meditation performed on each frequency-band deviation in dynamic PSD. We observed that many of these features significantly differed between concentrative and analytical sessions. Interestingly, these differences occurred mainly for the positive deviations from the baseline, Δ + _δ_ (*t* = 2.6883, df = 33, *p* = 0.0112), Δ + _θ_ (*t* = 4.4227, df = 33, *p* = 9.993e-05), Δ + _α1_ (*t* = 5.6642, df = 33, *p* = 2.595e-06), Δ + _α2_ (*t* = 2.962, df = 33, *p* = 0.0056), Δ + _α3_ (*t* = 2.5253, df = 33, *p* = 0.0165), Δ + _β1_ (*t* = 4.2475, df = 33, *p* = 1.6581e-03), Δ + _β2_ (*t* = 2.9086, df = 33, *p* = 6.4496e-04) compared to the negative deviations; Δ-_α1_(*t* = −2.2365, df = 33, *p* = 0.0322). Particularly, except for Δ-α1, all these features showed higher values during concentrative meditation compared to analytical meditation. In Figure 4, we separately report the maximum variation of the α peak (i.e., Δ_α-peak_). This feature, which is directly observable only in CHR PSD analysis, was significantly different between conditions (*t* = 4.9434, df = 33, *p* = 2.1795e-05) and seems to markedly distinguish between the two types of meditation compared to the other considered features.

Overall, since these features represent a variation between the meditation conditions and the resting baseline, this result indicates that concentrative sessions elicited stronger variations in the EEG power compared to analytical sessions.

### 3.3 Peaks in PSD in beta frequency range

**Figure 5:**
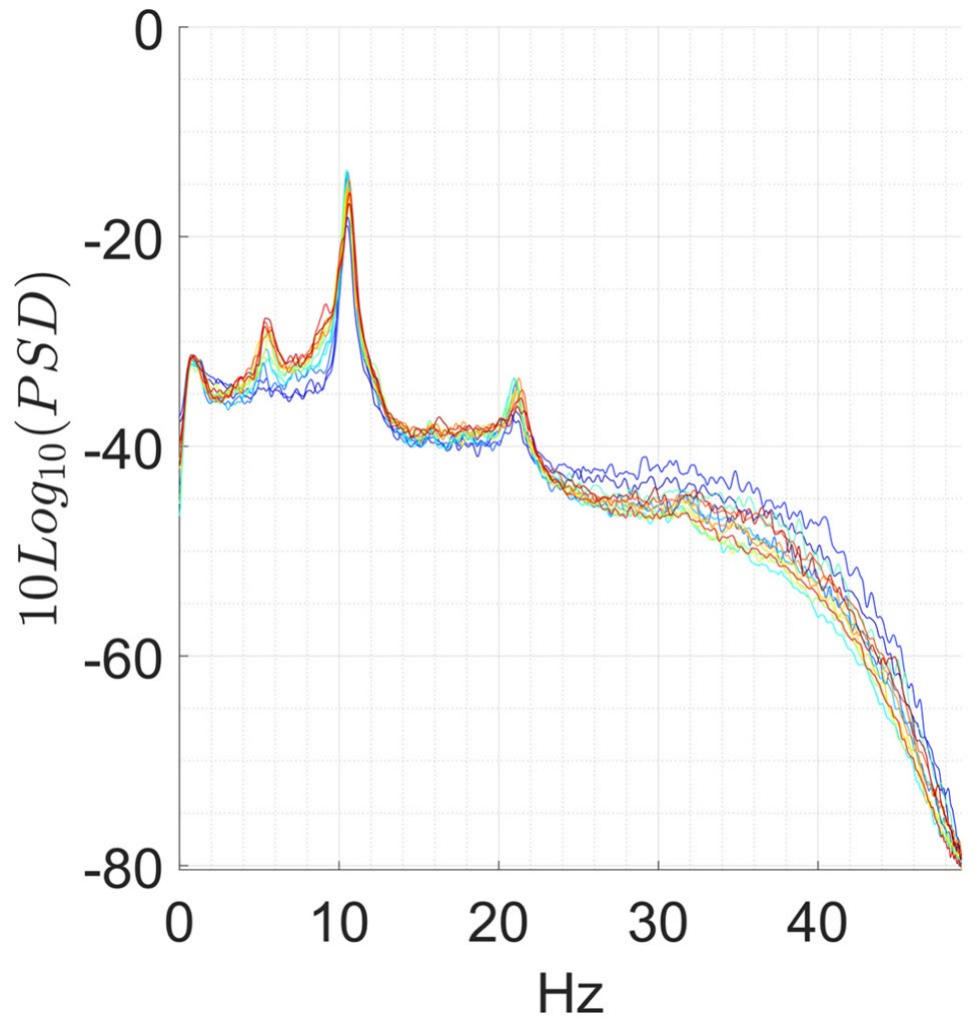
Exemplary of *bump*. A marked peak is observed at 21.5 Hz.

We found several EEG sessions for which a marked peak (*bump*) in the spectrum in the β range was present. *Bumps*, which sometimes increased up to 6 dB during the session, were clearly observed in 20 sessions out of 35. An exemplary of this phenomenon is reported in Figure 5, while in the Supplementary materials (Table_a_supp_mat.xlsx), we report the presence/absence of *bumps* during baseline and meditation, as well as their PSD difference (Δ*_bump_*) and the frequency at which they occurred. The *bump* was more frequent in the advanced meditators (13/16), compared to the beginners (2/9) and more frequent during concentrative sessions (12/16), compared to analytical (8/19).

### 3.4 Special cases

**Figure 6:**
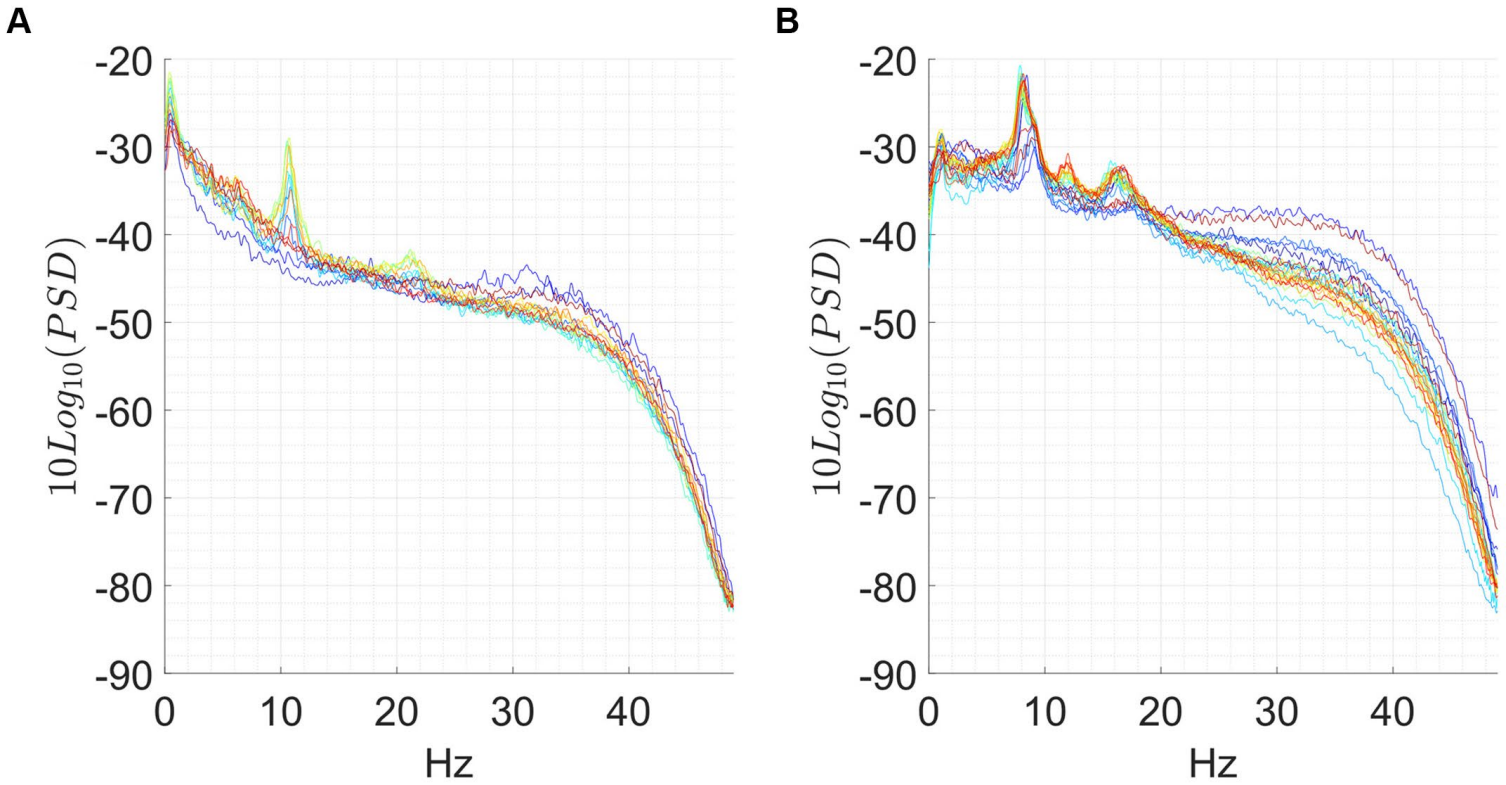
**(A)** In this subject the α peak is absent on the baseline. Then it emerges at frame 55–60 (green line), to turn off abruptly at frame 65–70 and remain off until the end of the session. **(B)** In this subject the α peak, is reduced in the baseline and then there is a first switch in frame 10–15 in which the whole spectrum grows, and the alpha peak is absorbed, then the spectrum returns to the state prior to the switch, and we assist to an increase of the alpha peak for approximately 95 min. In windows ranging from 110 to 115 min the phenomenon happens again and the alpha peak disappears.

**Figure 7:**
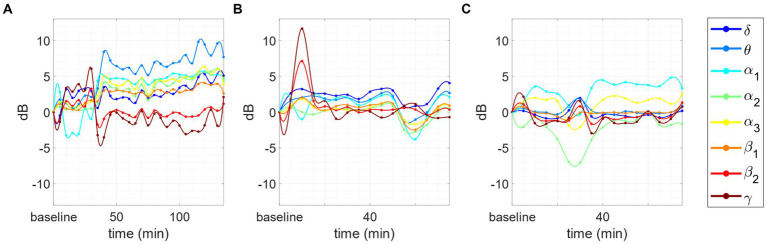
Changes in the PSD compared to baseline for 3 subjects. Inverse behavior between θ/α_1_/α_2_ and β_2_/γ AFB PSD. **(A)** After the first part of the session the power in α_1_ and α_2_ increase with respect to the baseline, whereas β_2_ and γ decrease: the phenomenon is evident in the first 7 points (35 min). **(B)** The inverted trend starts after 10 min of meditation and continues for the whole session: the phenomenon is evident in the first 8 points (40 min). **(C)** The inverted trend is clear after at the very beginning of the session (10 to 20 min) and during the las 20 min of meditation: the inverse behavior is evident in the initial and final peaks situated, respectively, between 10 and 20 min and 55 and 70 min.

In addition to these widespread differences that characterized the two types of meditation, we observed some isolated events, which may be of interest and which, in our opinion, deserve a phenomenological description. These phenomena have been observed only in concentrative sessions. Particularly, we observed that:

(i) In some meditators, the alpha peak in baseline does not exist.
(ii) In 3 cases, the α peak disappears abruptly after 10/15 min of concentration session, and then re-emerges later. This effect is accompanied by an abrupt variation of the spectrum in all bands. An exemplary is reported in Figure 6.
(iii) In other 3 cases, an inverse trend between the variations in the θ/α range and those in the β/γ range. More specifically, we observed that when AFB PSD increases in θ and/or α bands, a decrease in β/γ range is also present, and vice versa. This phenomenon happens just for advanced meditators during concentrative sessions. An example of this phenomenon is reported in Figure 7.

### 3.5 Effect of years of retreat on the maximum variation of the α peak

The effect of years of retreat of advanced meditators on the variation of the α peak showed a significant effect of “time_in_retreat” factor on such EEG neural correlate (*p* = 0.039). This result indicates that meditators whit more experience may exhibit more marked changes in the EEG signal, compared to less experienced ones.

**Figure 8:**
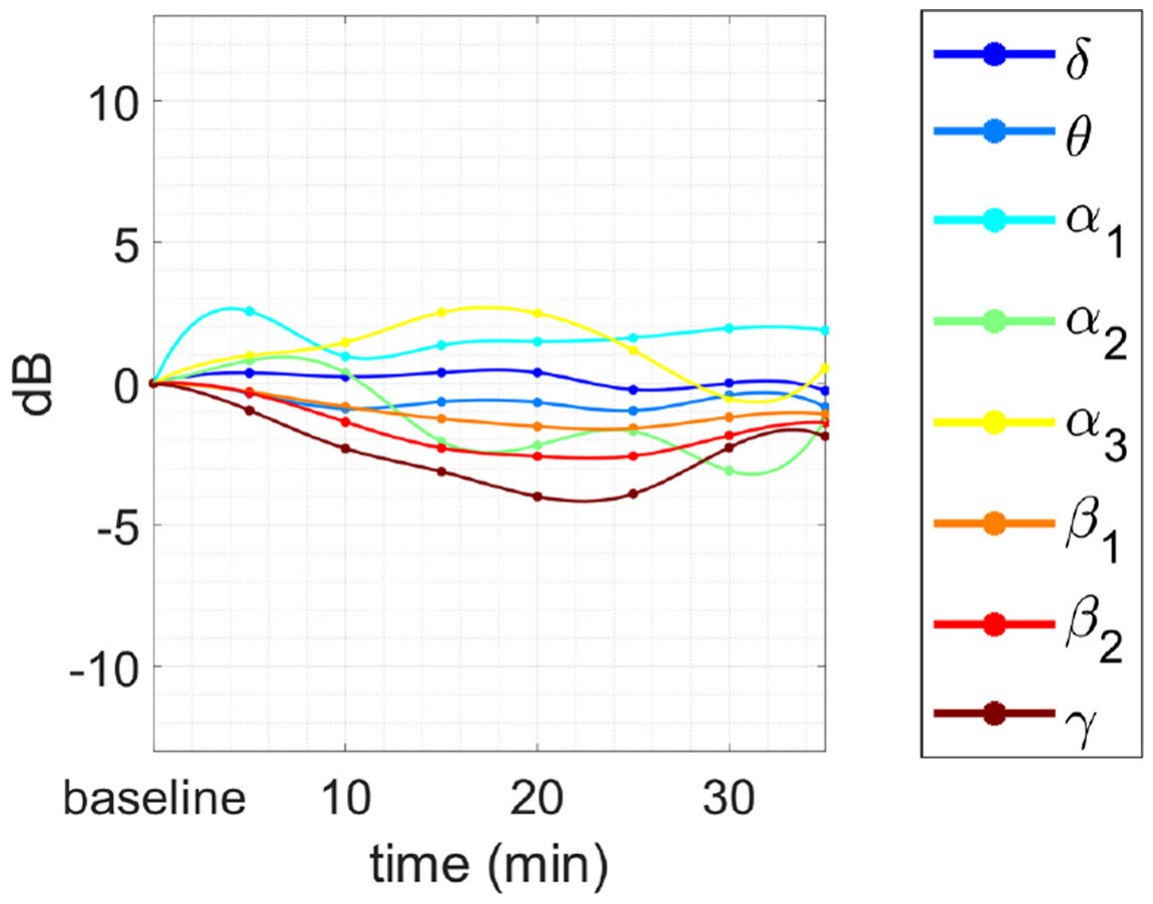
Changes in tvPSD compared to baseline. We report changes during an analytical session for the same meditator of Figure 7C.

This last phenomenon has been observed only in the case of intermediate or advanced meditators engaged in the concentrative session. Finally, the same meditator of Figure 6C engaged in an analytical session is reported in Figure 8. As we can see, this session does not show any negative correlation between the behavior of α_1_ and θ power with respect to those of β_2_ and γ power.

## 4 Discussion

In this work, we analyzed the neural correlates underpinning analytical and concentrative meditation. We compared several features derived from the EEG to identify the most specific electrophysiological correlates of each type of meditation. Twenty-three monks from the Tibetan Monastery of Sera Jey with different level of experience were analyzed during meditation sessions of variable duration. We showed that concentrative meditation exhibited the most relevant changes in the EEG features compared to the resting baseline. In addition, several significant differences in the EEG power features were observed between analytical and concentrative meditation. We analyzed a total of 35 meditation sessions (19 analytical, 16 concentrative) that allowed to test: (i) Whether the two main types of Tibetan meditation, the analytical and the concentrative one, are characterized by different neuronal correlates; (ii) If there are quantitative features derived from the EEG that can characterize the two types of meditation (i.e., that significantly differ between conditions).

We could exploit the unique opportunity of cooperating with a group of meditators that carried out their studies in the same community and shared the same cultural and social context for their last 10 years at least: i.e., the monks of the Sera Jey Tibetan Monastery in India. Our study provides some potential novel approaches for the study of meditation. Particularly, we aimed at limiting any potential influence of the experiment on the quality of each meditation session by adopting specific choices. Firstly, by acquiring the data inside the Tibetan Monastery of Sera Jey together with the volunteers and scholars of the Monastic University, we could examine the subjects in a more ecological fashion (i.e., without removing them from their own environment). This approach allowed to limit any potential influence of the typical laboratory setting on the meditation sessions, and thus on the acquired data (see Zaccaro et al., 2021 for a similar approach in a recent Nidra Yoga research). Secondly, we left the meditators to “do their best,” without any time limit on the meditation session. This choice was purposely made to account for subject specific differences in reaching the desired mental state or performing the desired meditation during the experiment. This latter aspect did not come without issues, since it heterogenized the nature of the acquisitions. To mitigate such an issue, we purposely set up the double strategy of performing both within session and between session analyses. The within session analysis allowed us to characterize the key aspects of each type of meditation, such as the variations in the spectra underpinning the meditation session. Instead, the between session analysis highlighted the most marked deviations from the resting baseline that, for different meditators, could occur at different instants during the meditation session. Particularly, we exploited not only averaged (i.e., AFB) but also High Resolution (i.e., CHR) PSD features to highlight these deviations that were further used to best discriminate between the two types of meditation. Overall, these choices offer interesting insights in the design of novel experiments that are naturally influenced by subjective differences.

### 4.1 Concentrative vs. analytical meditation

Concentrative meditation elicited variations in the EEG power compared to baseline that differed from those elicited by analytical meditation. Particularly, Δ_+δ_, Δ_+θ_, Δ_+α1_, Δ_+α2_, Δ_+β1_, Δ_+β2_, Δ_-α1_ and Δ_α-peak_ were significantly different between the two practices. Positive deviations from the baseline (i.e., Δ_+δ_, Δ_+θ_, Δ_+α1_, Δ_+α2_, Δ_+β1_, Δ_+β2_), as well as Δ_α-peak_ were significantly higher for concentrative sessions, compared to analytical sessions. Conversely, the negative deviation observed for Δ_-α1_ was significantly lower during concentrative meditation compared to analytical meditation. These results indicate that the most evident changes in the EEG spectral properties between analytical and concentrative meditation happen in the form of an increased EEG power in low frequency range, mainly θ, α_1_, and α_2_, with respect to baseline, whereas a decrease in gamma range is observable in both type of sessions.

Among these features, Δ_α-peak_ is of particular interest, as it also handles the variation in the frequency of the alpha peak, which could vary during the session. Δ_α-peak_ corresponds to the maximum positive variation, during the session, of the height of the α peak with respect to the baseline, and it was the feature with the clearest differences between the two types of mediation. In this light, we may suggest that this feature is the most representative for distinguishing between analytical and concentrative meditation. Furthermore, from a visual inspection of the tvPSD, an increased α peak seems to be a hallmark of concentrative meditation that rarely appears in analytic sessions. Indeed, while for analytical sessions changes in PSD rarely exceed ~2 dB, concentrative ones showed much higher variations (up to 15 dB). This feature was further exploited to investigate for an effect of the years spent in retreat of advanced meditators on the EEG signal. For this subset, meditators are much comparable among them due to the rigorous scheme of their daily practice. Although preliminary, our results indicated a significant effect of years spent in retreat on the maximum variation of the α peak that would merit further investigation by enlarging the sample size used for the analysis.

In the field of Contemplative Neuroscience, the slowing of cortical EEG rhythms, specifically the augmentation of alpha and theta band power, predominantly across frontal regions (Fell et al., 2010), aligns with findings from systematic reviews on contemplative practices (Lomas et al., 2015; Lee et al., 2018). Modulation of alpha and theta band power constitutes a distinctive trait of non-ordinary states of consciousness induced through various means such as slow breathing, relaxation, hypnosis, and psychedelic substances (Aftanas and Golocheikine, 2001; Holroyd, 2003; Fumoto et al., 2004; Jacobs and Friedman, 2004; Yu et al., 2011; Park and Park, 2012; Timmermann et al., 2019), although these states have been effectively distinguished from meditation in numerous studies (Halsband et al., 2009; De Benedittis, 2015; Thompson and Markovic, 2016). At the first-person level, modulations of alpha and theta oscillations have been associated with positive psychological outcomes, including reduced levels of anxiety, depression, anger, and confusion, alongside heightened awareness, perceptual clarity, and introspective attention (Fumoto et al., 2004; Yu et al., 2011; Zaccaro et al., 2018, 2021). Furthermore, the enhancement of theta power has been correlated with the deepening of the meditative state and assumes a critical role in non-ordinary states of consciousness, as evidenced by recent studies on ultra-slow nasal stimulation and slow breathing practices (Piarulli et al., 2018; Zaccaro et al., 2021, 2022). The specific increase of alpha power during concentrative meditation in the present study may be elucidated in light of numerous prior studies exploring the interplay between alpha oscillations and inwardly directed attention regulation, sensory processing, and task performance. While conventionally interpreted as reflective of the brain’s “idle” state at rest, alpha oscillations are now acknowledged as active mechanisms in attentional modulation and excitability control (Ben-Simon et al., 2008). Alpha oscillations have been linked with heightened interoceptive attention, observed not only during meditation but also during other attentional tasks where participants internally shift focus, such as toward heartbeat sensations during cardiac interoceptive tasks, hence inhibiting external sources of distraction (Villena-González et al., 2017; Kritzman et al., 2022). Taking previous body of evidences into account (Foxe and Snyder, 2011; Hanslmayr et al., 2011; Diepen et al., 2019; Antonov et al., 2020), heightened alpha-band activity can be here interpreted as an index of an active inhibitory attentional mechanism, suppressing irrelevant and distracting inputs and reflecting the prioritization of task-relevant information (e.g., focusing and sustaining attention on a single object) over irrelevant stimuli, which is specifically required during concentrative meditation.

Of note, from a visual inspection of the tvPSD, we also observed that the most marked differences in the spectra of concentrative sessions occurred for those advanced meditators with more years of retreat. Interestingly, the duration of the session seemed to affect such variations. Indeed, the most marked variations were observed for those sessions whose duration exceeded 45 min. However, considering the limited size of the sample, we could not properly test the influence of these features in a more rigorous way. In this light, feature improvements would include a larger number of subjects for each group in the analysis.

Although preliminary, our results seem to indicate that expert meditators engaged in concentrative sessions show marked and repeatable modifications of the EEG power to hypothesize a non-ordinary state of consciousness different from wakefulness and sleep at least as far as neural correlates observable by EEG is concerned. This is particularly true in the case of advanced meditators in retreat, whose PSD during the baseline can be completely modified in all the bands during the meditation session (see Figure 1B). Further support to this claim is given by the fact that equally experienced full-time meditators did not show equally significant changes in power during analytical sessions (see Figure 1A and Supplementary materials – single_subject_ranksum_tests_results.zip and single_subjects_tvPSD.zip-). However, we cannot exclude that analytical meditation may induce alterations in brain activity not properly captured by power measures. In this light, future studies involving other measures, such as connectivity and network metrics may help at understanding the underpinnings of this practice.

### 4.2 Special cases

In some participants, we observed a series of phenomena that may be of particular interest. Probably, the most relevant is the presence of *bumps*: i.e., the presence of a marked peak in the β frequency range observable only from CHR PSD analysis. This phenomenon occurred more frequently for advanced meditators in both analytical and concentrative sessions (13/15) compared to beginners (2/9). In this light, their presence could represent a trait of experience rather than of type of meditation. Yet, considering that experienced meditators performed mostly concentrative sessions, while beginners performed mostly analytical sessions, we cannot completely support this hypothesis that merits further investigation. However, since such a phenomenon has never been reported in the literature of meditation, we provide here a first observational description. Jensen and Mazaheri (2010) suggested that high-α/low-β controls information processing by inhibiting task-irrelevant regions in the brain. In this light, we could hypothesize that experienced meditators are more able to perform such an inhibition compared to beginners.

Another interesting behavior is the abrupt commutation of the PSD trend with abrupt disappearance and subsequent reappearance of the peak in the α band. While this behavior is very common when closing the eyes (i.e., switching from eyes-open to eyes-closed condition), it is less evident in stationary eyes-closed condition. We speculate that this phenomenon may reflect a switching between different mental states. However, more investigation is needed in this direction. For instance, it is suggested to include in the experimental protocol a system for reporting potential switching in mental states experienced by the meditator, as for instance a trigger button manually annotate on the EEG acquisition particular events experienced during the session. Moreover, we cannot exclude that using shorter time windows such changes may be smoother.

Finally, based on the AFB PSD analysis, we observed, in some cases, an opposite behavior between the time-dependent variations of PSD in the θ/α1 bands and those in the β2/γ bands during concentrative sessions, in the sense that when one increased the other decreased and vice versa. Interestingly, this happened for advanced meditators but not for intermediate meditators nor beginners. Considering that concentrative sessions are performed mostly by intermediate or advanced meditators, it may be suggested to include a larger population of advanced meditators for further testing on this phenomenon. In this light, given the coupling properties of θ/γ frequency bands future analysis should focus on the study of brain connectivity which may provide complementary information to the one provided in this work.

### 4.3 Future developments

Although our results are based on robust statistical testing, they may require further refinements of the data acquisition and processing procedure. Particularly, it will be necessary to increase the number of volunteers, as well as to balance their number among beginners, intermediate and advanced in both conditions (i.e., concentrative and analytical). This aspect is particularly important since, in the investigated sample, there is an intrinsic correlation between the meditator experience and the type of meditation performed. More specifically, advanced meditators tend to perform concentrative sessions, compared to beginners who focused more on analytic meditation. In this light, we cannot exclude that some of the observed differences between concentrative and analytic meditation may be also due to meditator’s expertise. However, it is worth noting that from single subject analyses (see Supplementary material) analytic sessions are similar among them, independently from the level of expertise of the meditator. Accordingly, it is reasonable to assume that the observed changes in the neural correlates of concentrative and analytical meditation may derive from intrinsic differences in the type of meditation rather than on meditator’s experience.

Additionally, it would be particularly useful to integrate in the analysis the information reported in the questionnaire submitted to the volunteer before and after the meditation session, as well as to include in the analysis the information on the meditator’s subjective experience during the various phases of the session. In this work, we assumed that being analytic and concentrative meditation two completely different practices, differences in the EEG neural correlates would be observable even based on such simple distinction. Nevertheless, more sophisticated analysis could include subjective experience of meditators by including specific descriptions of the meditative session and state through the use of structured questionnaires. This latter kind of integration would be beneficial for enhancing the psychophysiological interpretations of our results, which have been left for future studies.

## Data availability statement

The datasets presented in this article are not readily available because they contain information that could compromise the privacy of research participants. Requests to access the datasets should be directed to bruno.neri@unipi.it, alejandro.callara@unipi.it.

## Ethics statement

The studies involving humans were approved by Ethical Committee of the University of Pisa (n. 0117745/2020). The studies were conducted in accordance with the local legislation and institutional requirements. The participants provided their written informed consent to participate in this study.

## Author contributions

BN: Conceptualization, Data curation, Formal analysis, Funding acquisition, Investigation, Methodology, Project administration, Resources, Software, Supervision, Validation, Visualization, Writing – original draft, Writing – review & editing. AC: Conceptualization, Data curation, Formal analysis, Investigation, Methodology, Software, Supervision, Validation, Visualization, Writing – original draft, Writing – review & editing. NV: Conceptualization, Formal analysis, Investigation, Methodology, Writing – review & editing. DM: Conceptualization, Investigation, Methodology, Writing – review & editing, Formal analysis. AZ: Conceptualization, Formal analysis, Investigation, Methodology, Writing – original draft, Writing – review & editing. AP: Formal analysis, Writing – review & editing. ML: Formal analysis, Software, Writing – review & editing. NN: Conceptualization, Formal analysis, Investigation, Writing – original draft. JK: Conceptualization, Formal analysis, Investigation, Writing – original draft, Writing – review & editing. NS: Conceptualization, Formal analysis, Investigation, Writing – original draft, Writing – review & editing. AG: Conceptualization, Formal analysis, Funding acquisition, Investigation, Methodology, Project administration, Resources, Supervision, Writing – review & editing.

## References

[ref1] Aftanas L. I. Golocheikine S. A. (2001). Human anterior and frontal midline theta and lower alpha reflect emotionally positive state and internalized attention: high-resolution EEG investigation of meditation. Neurosci. Lett. 310, 57–60. doi: 10.1016/S0304-3940(01)02094-8, PMID: 11524157

[ref2] Antonov P. A. Chakravarthi R. Andersen S. K. (2020). Too little, too late, and in the wrong place: alpha band activity does not reflect an active mechanism of selective attention. Neuroimage 219:117006. doi: 10.1016/j.neuroimage.2020.117006, PMID: 32485307

[ref4] Benjamini Y. Yekutieli D. (2001). The control of the false discovery rate in multiple testing under dependency. Ann. Stat. 29, 1165–1188. doi: 10.1214/aos/1013699998

[ref5] Ben-Simon E. Podlipsky I. Arieli A. Zhdanov A. Hendler T. (2008). Never resting brain: simultaneous representation of two alpha related processes in humans. PLoS One 3:e3984. doi: 10.1371/journal.pone.0003984, PMID: 19096714 PMC2602982

[ref6] Billeci L. Callara A. L. Guiducci L. Prosperi M. Morales M. A. Calderoni S.. (2023). A randomized controlled trial into the effects of probiotics on electroencephalography in preschoolers with autism. Autism 27, 117–132. doi: 10.1177/13623613221082710, PMID: 35362336 PMC9806478

[ref7] Brewer J. A. Worhunsky P. D. Gray J. R. Tang Y.-Y. Weber J. Kober H. (2011). Meditation experience is associated with differences in default mode network activity and connectivity. Proc. Natl. Acad. Sci. 108, 20254–20259. doi: 10.1073/pnas.1112029108, PMID: 22114193 PMC3250176

[ref8] Buckner R. L. Carroll D. C. (2007). Self-projection and the brain. Trends Cogn. Sci. 11, 49–57. doi: 10.1016/j.tics.2006.11.004 17188554

[ref9] Cahn B. R. Polich J. (2006). Meditation states and traits: EEG, ERP, and neuroimaging studies. Psychol. Bull. 132, 180–211. doi: 10.1037/0033-2909.132.2.180, PMID: 16536641

[ref10] Callara A. L. Morelli M. S. Hartwig V. Landini L. Giannoni A. Passino C.. (2020). Ld-EEG effective brain connectivity in patients with Cheyne-stokes respiration. IEEE Trans. Neural Syst. Rehabil. Eng. 28, 1216–1225. doi: 10.1109/TNSRE.2020.2981991, PMID: 32191895

[ref11] Dahl C. J. Lutz A. Davidson R. J. (2015). Reconstructing and deconstructing the self: cognitive mechanisms in meditation practice. Trends Cogn. Sci. 19, 515–523. doi: 10.1016/j.tics.2015.07.001, PMID: 26231761 PMC4595910

[ref12] Dalai Lama. (1995). The Dalai Lama’s book of wisdom. S.D. Available at: https://www.goodreads.com/book/show/104957.The_Dalai_Lama_s_Book_of_Wisdom.

[ref3] De Benedittis G. (2015). Neural mechanisms of hypnosis and meditation. J. Physiol. 109, 152–164. doi: 10.1016/j.jphysparis.2015.11.001, PMID: 26554845

[ref14] Delorme A. Makeig S. (2004). EEGLAB: an open source toolbox for analysis of single-trial EEG dynamics including independent component analysis. J. Neurosci. Methods 134, 9–21. doi: 10.1016/j.jneumeth.2003.10.009, PMID: 15102499

[ref15] Diepen V. Rosanne M. Foxe J. J. Mazaheri A. (2019). The functional role of alpha-band activity in attentional processing: the current zeitgeist and future outlook. *Curr. Opin. Psychol.* Attent. Percept. 29, 229–238. doi: 10.1016/j.copsyc.2019.03.015, PMID: 31100655

[ref16] Farb N. Daubenmier J. Price C. J. Gard T. Kerr C. Dunn B. D.. (2015). Interoception, contemplative practice, and health. Front. Psychol. 6:763. doi: 10.3389/fpsyg.2015.00763, PMID: 26106345 PMC4460802

[ref17] Fell J. Axmacher N. Haupt S. (2010). From alpha to gamma: electrophysiological correlates of meditation-related states of consciousness. Med. Hypotheses 75, 218–224. doi: 10.1016/j.mehy.2010.02.025, PMID: 20227193

[ref18] Fox K. C. R. Dixon M. L. Nijeboer S. Girn M. Floman J. L. Lifshitz M.. (2016). Functional neuroanatomy of meditation: a review and meta-analysis of 78 functional neuroimaging investigations. Neurosci. Biobehav. Rev. 65, 208–228. doi: 10.1016/j.neubiorev.2016.03.021, PMID: 27032724

[ref19] Fox K. C. R. Nijeboer S. Dixon M. L. Floman J. L. Ellamil M. Rumak S. P.. (2014). Is meditation associated with altered brain structure? A systematic review and meta-analysis of morphometric neuroimaging in meditation practitioners. Neurosci. Biobehav. Rev. 43, 48–73. doi: 10.1016/j.neubiorev.2014.03.016, PMID: 24705269

[ref20] Fox Kieran C. R. Rael Cahn B.. (2021). «Meditation and the brain in health and disease». In The Oxford handbook of meditation, Miguel Farias Cura di Brazier David Lalljee Mansur, ed. Oxford: Oxford University Press.

[ref21] Foxe J. J. Snyder A. C. (2011). The role of alpha-band brain oscillations as a sensory suppression mechanism during selective attention. Front. Psychol. 2:154. doi: 10.3389/fpsyg.2011.00154, PMID: 21779269 PMC3132683

[ref22] Fumoto M. Sato-Suzuki I. Seki Y. Mohri Y. Arita H. (2004). Appearance of high-frequency alpha band with disappearance of low-frequency alpha band in EEG is produced during voluntary abdominal breathing in an eyes-closed condition. Neurosci. Res. 50, 307–317. doi: 10.1016/j.neures.2004.08.005, PMID: 15488294

[ref23] Halsband U. Mueller S. Hinterberger T. Strickner S. (2009). Plasticity changes in the brain in hypnosis and meditation. Contemp. Hypn. 26, 194–215. doi: 10.1002/ch.386

[ref24] Hanslmayr S. Gross J. Klimesch W. Shapiro K. L. (2011). The role of alpha oscillations in temporal attention. Brain Res. Rev. 67, 331–343. doi: 10.1016/j.brainresrev.2011.04.002 21592583

[ref25] Holroyd J. (2003). The science of meditation and the state of hypnosis. Am. J. Clin. Hypn. 46, 109–128. doi: 10.1080/00029157.2003.10403582 14609297

[ref26] Jacobs G. D. Friedman R. (2004). EEG spectral analysis of relaxation techniques. Appl. Psychophysiol. Biofeedback 29, 245–254. doi: 10.1007/s10484-004-0385-2, PMID: 15707254

[ref27] Jensen O. Mazaheri A. (2010). Shaping functional architecture by oscillatory alpha activity: gating by inhibition. Front. Hum. Neurosci. 4:186. doi: 10.3389/fnhum.2010.00186, PMID: 21119777 PMC2990626

[ref28] Jerath R. Crawford M. W. Barnes V. A. Harden K. (2015). Self-regulation of breathing as a primary treatment for anxiety. Appl. Psychophysiol. Biofeedback 40, 107–115. doi: 10.1007/s10484-015-9279-8, PMID: 25869930

[ref29] Jiang H. He B. Xiaoli Guo X. Wang M. G. Wang Z. Xue T.. (2020). Brain–heart interactions underlying traditional Tibetan Buddhist meditation. Cereb. Cortex 30, 439–450. doi: 10.1093/cercor/bhz095, PMID: 31163086

[ref30] Kabat-Zinn J. (1991). Full catastrophe living: Using the wisdom of your body and mind to face stress, pain, and illness. New York, NY: Delta Trade Paperbacks.

[ref31] Kritzman L. Eidelman-Rothman M. Keil A. Freche D. Sheppes G. Levit-Binnun N. (2022). Steady-state visual evoked potentials differentiate between internally and externally directed attention. Neuroimage 254:119133. doi: 10.1016/j.neuroimage.2022.119133, PMID: 35339684

[ref32] Lee D. J. Kulubya E. Goldin P. Goodarzi A. Girgis F. (2018). Review of the neural oscillations underlying meditation. Front. Neurosci. 12:178. doi: 10.3389/fnins.2018.00178 29662434 PMC5890111

[ref33] Lomas T. Ivtzan I. Fu C. H. Y. (2015). A systematic review of the neurophysiology of mindfulness on EEG oscillations. Neurosci. Biobehav. Rev. 57, 401–410. doi: 10.1016/j.neubiorev.2015.09.018, PMID: 26441373

[ref34] Lott D. T. Tenzin Yeshi N. Norchung S. D. Tsering N. Jinpa N. Woser T.. (2020). No detectable electroencephalographic activity after clinical declaration of death among Tibetan Buddhist meditators in apparent Tukdam, a putative postmortem meditation state. Front. Psychol. 11:599190. doi: 10.3389/fpsyg.2020.599190, PMID: 33584435 PMC7876463

[ref35] Lutz A. Slagter H. A. Dunne J. D. Davidson R. J. (2008). Attention regulation and monitoring in meditation. Trends Cogn. Sci. 12, 163–169. doi: 10.1016/j.tics.2008.01.005, PMID: 18329323 PMC2693206

[ref36] Medvedev S. V. Boytsova J. A. Bubeev Y. A. Kaplan A. Y. Kokurina E. V. Olsen A.. (2022). Traditional Buddhist meditations reduce mismatch negativity in experienced monk- practitioners. Int. J. Psychophysiol. 181, 112–124. doi: 10.1016/j.ijpsycho.2022.08.011, PMID: 36057406

[ref37] Mehrmann C. Karmacharya R. (2013). Principles and neurobiological correlates of concentrative, diffuse, and insight meditation. Harv. Rev. Psychiatry 21, 205–218. doi: 10.1097/HRP.0b013e31828e8ef4, PMID: 24651509

[ref38] Mullen T. R. Kothe C. A. E. Chi Y. M. Ojeda A. Kerth T. Makeig S.. (2015). Real-time neuroimaging and cognitive monitoring using wearable dry EEG. I.E.E.E. Trans. Biomed. Eng. 62, 2553–2567. doi: 10.1109/TBME.2015.2481482, PMID: 26415149 PMC4710679

[ref39] Nash J. D. Newberg A. Awasthi B. (2013). Toward a unifying taxonomy and definition for meditation. Front. Psychol. 4:806. doi: 10.3389/fpsyg.2013.00806, PMID: 24312060 PMC3834522

[ref40] Palmer J. Kreutz-Delgado K. Makeig S.. (2011). AMICA: an adaptive mixture of independent component analyzers with shared components. Available at: https://www.semanticscholar.org/paper/AMICA-%3A-An-Adaptive-Mixture-of-Independent-with-Palmer-Kreutz-Delgado/5774e96ad450c228400dc311f16caf1f20967c10.

[ref41] Park Y.-J. Park Y.-B. (2012). Clinical utility of paced breathing as a concentration meditation practice. Complement. Ther. Med. 20, 393–399. doi: 10.1016/j.ctim.2012.07.008, PMID: 23131369

[ref42] Piarulli A. Zaccaro A. Laurino M. Menicucci D. De Vito A. Bruschini L.. (2018). Ultra-slow mechanical stimulation of olfactory epithelium modulates consciousness by slowing cerebral rhythms in humans. Sci. Rep. 8:6581. doi: 10.1038/s41598-018-24924-9, PMID: 29700421 PMC5919905

[ref43] Raichle M. E. (2015). The Brain’s default mode network. Annu. Rev. Neurosci. 38, 433–447. doi: 10.1146/annurev-neuro-071013-014030 25938726

[ref44] Tang Y.-Y. Hölzel B. K. Posner M. I. (2015). The neuroscience of mindfulness meditation. Nat. Rev. Neurosci. 16, 213–225. doi: 10.1038/nrn3916 25783612

[ref45] Thompson Evan Markovic Jelena. (2016). Hypnosis and meditation: a neurophenomenological comparison. Consultato 2024. Available at: https://www.academia.edu/29286671/Hypnosis_and_meditation_a_neurophenomenological_comparison.

[ref46] Timmermann C. Roseman L. Schartner M. Milliere R. Williams L. T. J. Erritzoe D.. (2019). Neural correlates of the DMT experience assessed with multivariate EEG. Sci. Rep. 9:16324. doi: 10.1038/s41598-019-51974-4, PMID: 31745107 PMC6864083

[ref47] Urigüen J. A. Garcia-Zapirain B. (2015). EEG artifact removal-state-of-the-art and guidelines. J. Neural Eng. 12:031001. doi: 10.1088/1741-2560/12/3/031001, PMID: 25834104

[ref48] Vago D. R. Silbersweig D. A. (2012). Self-awareness, self-regulation, and self-transcendence (S-ART): a framework for understanding the neurobiological mechanisms of mindfulness. Front. Hum. Neurosci. 6:296. doi: 10.3389/fnhum.2012.00296, PMID: 23112770 PMC3480633

[ref13] Van Dam N. T. van Vugt M. K. Vago D. R. Schmalzl L. Saron C. D. Olendzki A.. (2018). Mind the hype: a critical evaluation and prescriptive agenda for research on mindfulness and meditation. Perspect. Psychol. Sci. 13, 36–61. doi: 10.1177/1745691617709589, PMID: 29016274 PMC5758421

[ref49] van Vugt M. K. Pollock J. Johnson B. Gyatso K. Norbu N. Lodroe T.. (2020). Inter-brain synchronization in the practice of Tibetan monastic debate. Mindfulness 11, 1105–1119. doi: 10.1007/s12671-020-01338-1

[ref50] Vieten C. Helané Wahbeh B. Cahn R. MacLean K. Estrada M. Mills P.. (2018). Future directions in meditation research: recommendations for expanding the field of contemplative science. PLoS One 13:e0205740. doi: 10.1371/journal.pone.0205740, PMID: 30403693 PMC6221271

[ref51] Villena-González M. Moënne-Loccoz C. Lagos R. A. Alliende L. M. Billeke P. Aboitiz F.. (2017). Attending to the heart is associated with posterior alpha band increase and a reduction in sensitivity to concurrent visual stimuli. Psychophysiology 54, 1483–1497. doi: 10.1111/psyp.12894, PMID: 28560781

[ref52] Wallace B. A. (1999). The Buddhist tradition of Samatha: methods for refining and examining consciousness. J. Conscious. Stud. 6, 175–187,

[ref53] Wallace B. A. (2006). The attention revolution: unlocking the power of the focused mind. New York: Simon and Schuster.

[ref54] Yu X. Fumoto M. Nakatani Y. Sekiyama T. Kikuchi H. Seki Y.. (2011). Activation of the anterior prefrontal cortex and serotonergic system is associated with improvements in mood and EEG changes induced by Zen meditation practice in novices. Int. J. Psychophysiol. 80, 103–111. doi: 10.1016/j.ijpsycho.2011.02.004, PMID: 21333699

[ref55] Zaccaro A. Piarulli A. Laurino M. Garbella E. Menicucci D. Neri B.. (2018). How breath-control can change your life: a systematic review on psycho-physiological correlates of slow breathing. Front. Hum. Neurosci. 12:353. doi: 10.3389/fnhum.2018.00353, PMID: 30245619 PMC6137615

[ref56] Zaccaro A. Piarulli A. Melosini L. Menicucci D. Gemignani A. (2022). Neural correlates of non-ordinary states of consciousness in pranayama practitioners: the role of slow nasal breathing. Front. Syst. Neurosci. 16:803904. doi: 10.3389/fnsys.2022.803904, PMID: 35387390 PMC8977447

[ref57] Zaccaro A. Riehl A. Piarulli A. Alfì G. Neri B. Menicucci D.. (2021). The consciousness state of traditional Nidrâ yoga/modern yoga Nidra: phenomenological characterization and preliminary insights from an EEG study. Int. J. Yoga Ther. 31:14. doi: 10.17761/2021-D-20-00014 34727178

