# Supplementary material for: Report from a Tibetan Monastery: EEG neural correlates of concentrative and analytical meditation
Source: Front Psychol. 2024 May 2;15:1348317. doi: 10.3389/fpsyg.2024.1348317 (PMC11098278; doi:10.3389/fpsyg.2024.1348317)
Supplement: Supplementary file 3 [file Table_1.DOCX]

Supplementary Material

# Questionnaire

**Profile of the meditator**

**First name**: ……………. **Second name**:… ………..**Date of Birth**: __/__/____

**Short bio:**

………………………………………………………………………………………………………………………………………………………………………………………………………………………………………………………………………………………………………………………………………………………………………………………………………………………..….…………………………………………………………………………………………………………………………………………………………………………………………………………………………………………………………………

**Q1.** When did you begin to meditate?

**Q2.** Since then, did you practice in a continuous way?

**Q3.** How many hours/minutes do you practice every day?

**Q4.** Did you never stay in a retreat?

**Q5.** If yes, how many years?

**Notes:** …………………………………………………………………………………………………………………………………………………………………………….………………………………………………………………………………………………………………………………………………………………..

Put a cross in the relevant boxes concerning system (**Sutra/Tantra**) and typology (**Analytical/Concentrative**)

|  | **SUTRA SYSTEM** | | **TANTRA SYSTEM** | | **NOTES** |
| --- | --- | --- | --- | --- | --- |
| **ANALITYCAL** | LAMRIM |  |  |  |  |
|  | EMPITINESS |  |  |  |  |
|  | COMPASSION |  |  |  |  |
|  |  |  |  |  |  |
|  |  |  |  |  |  |
|  |  |  |  |  |  |
|  | | |  | |  |
| **CONCENTRATIVE** | BREATH |  | GENER. STAGE |  |  |
|  | VISUALIZATION |  | COMPL. STAGE |  |  |
|  | NATURE OF MIND |  | DZOGCHEN |  |  |
|  | EMPTINESS |  | DZOGRIN |  |  |
|  | MANTRA |  | MAHAMUDRA |  |  |
|  |  |  |  |  |  |

**Notes**:……………………………………………………………………………………………………………………………………………………………………………………………………………………………………………………………………………………………………………………………………………………………………………………………………………………………………………………………………………………………………………………………………………………………………………………………………………………………………………………………………………………………………………………………………………………………………………………………………………………………………………………………

**Meditation session measurements**

**Date:** __/__/____ **Time:**

**First name**: ____________ **Second name**: ___________ **Date of Birth**: __/__/____

**Place of the acquisition:**

**Preparation starts at**: **Finish at**:

**Acquisition System**:

**Syncronization with GPS time mark?** YES/NO

**Notes on electrodes impedence:**

**Acquisition starts at**:

**Basal** (eyes-closed, relaxes, no meditation) **starts at**: **finish at:**

**Typology of meditation:**

**Q5.** Is meditation session composed by different phases?

**Q6.** If Yes, which phases? ………………………………………………………………………………………………………………………………………………………………………………………………………………………………………………………………………………………………………………………………………………

**Q7.** The meditator has been asked to push the button for at least 3 second at each phase change? Yes/No

**Q8.** The meditator has been asked to push the button at the beginning and at the end of the session? Yes/No

**Notes on the session:** ..………………………………………………………………………………………………………………………………….

**Check of electrodes impedence at end of the session:** …………………………………………………………………………………

**Final notes:** ……………………………………………………………………………………………………………………………………………………

………………………………………………………………………………………………………………………………………………………………………………………………………………………………………………………………………………………………………………………………………………………………………………………………………………………………………………………………………………………………………………………………………………………………………………………………………………………………………………………………………………………………………………………………………………………………………………………………………………………………………………………………………

**Experimenter:** ………………………………………………………………………………………………………………………………………………
